# Mullerian tumor masquerading as cutaneous breast cancer

**DOI:** 10.1097/JW9.0000000000000219

**Published:** 2025-07-22

**Authors:** Grace C. Herron, Alexandra C. Hristov, Richard L. Cantley, Elisabeth A. Pedersen

**Affiliations:** a Department of Dermatology, University of Michigan Medical School, Ann Arbor, Michigan; b Department of Dermatology, University of Michigan Medical Center, Ann Arbor, Michigan; c Department of Pathology, University of Michigan Medical Center, Ann Arbor, Michigan

**Keywords:** breast cancer, cutaneous metastasis, Mullerian tumor

**What is known about this subject in regard to women and their families?**

- Breast and gynecologic cancers cause major health issues in women and occasionally present with metastasis to the skin.
- When skin metastases arise, it can be challenging to determine the source of metastasis, as some gynecological cancers present with hormone receptor profiles that may be indistinguishable from breast cancer.

**What is new from this article as messages for women and their families?**

- Gynecologic cancers can metastasize to the breast. These malignancies can mimic primary, recurrent, or metastatic breast cancer, thus causing diagnostic challenges and potential delays in treatment.
- This case highlights a unique presentation of metastatic Mullerian carcinoma initially presenting in the breast and highlights that skin metastases may be the first presentation of an internal malignancy.
- This patient’s case was complicated by a prior history of breast cancer, thus required additional diagnostic testing to discern the origin of the metastases to the breast.
- In addition to breast cancer, dermatologists might consider including gynecologic malignancy on their differential diagnosis when approaching lesions suspicious for cutaneous metastases arising in the skin of the breast.

## Introduction

Cutaneous metastases to the breast are rare and most commonly occur from a primary breast carcinoma.^1^ While ovarian and peritoneal carcinomas typically metastasize within the peritoneum and to local lymph nodes, they may also rarely metastasize to the breast skin and tissue.^2,3^ In these cases, distinguishing the source of metastasis to pursue appropriate treatment can be challenging, as both breast and gynecological cancers may present with hormone receptor positivity. Here, we report a patient with a remote history of breast cancer presenting with a breast rash as the initial presentation of a new Mullerian carcinoma.

## Case report

An 89-year-old woman presented with a 1-month history of an asymptomatic bilateral breast rash. History was pertinent for stage I estrogen receptor (ER) positive, progesterone receptor (PR) positive, and human epidermal growth factor receptor 2 (HER2) negative invasive ductal carcinoma of the breast over 15 years ago. She was treated with lumpectomy, radiation, and tamoxifen, and remained in remission. Her last mammogram, 8 months prior, was normal.

**Fig. 1.**
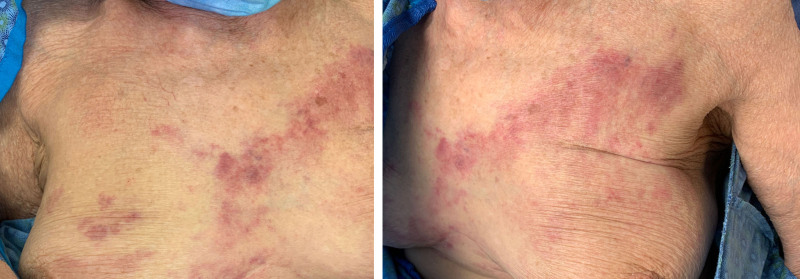
Erythematous to violaceous, ill-defined indurated plaques scattered across the breasts and chest.

**Fig. 2.**
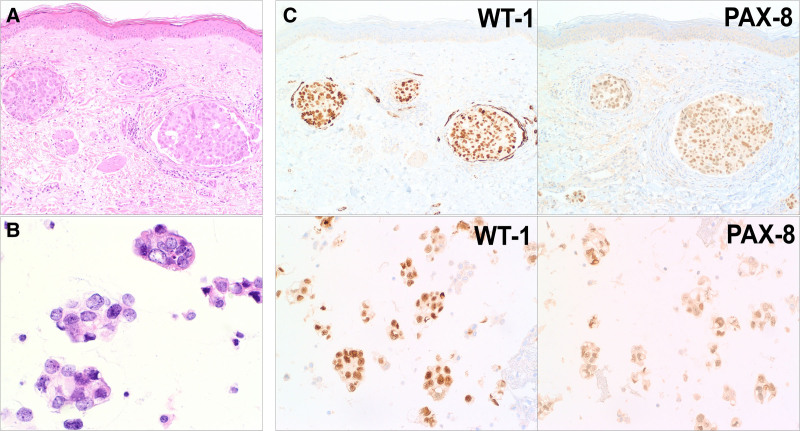
Pathology and immunohistochemical analyses. (A) Histopathologic findings of breast punch biopsy specimen revealing frankly-malignant, cohesive cells within angiolymphatic spaces (100×). (B) Cytopathology of ascites fluid showed similar appearing malignant cells (200×). (C) Cells in both the breast biopsy (upper panels, 100×) and ascites cytology (lower panels, 200×) expressed characteristic Mullerian tumor markers Wilms’ tumor 1 and paired-box gene 8.

Erythematous-to-violaceous ill-defined thin plaques were scattered across the breasts (Fig. 1). The appearance raised concern for breast cancer recurrence or radiation-induced angiosarcoma. Biopsy from the left breast skin showed cohesive cells within angiolymphatic spaces that were morphologically compatible with intralymphatic carcinoma (Fig. 2A). These cells were ER negative (<1%), PR positive (40%), and HER2 positive (3+). Computed tomography imaging of the chest, abdomen, and pelvis revealed large volume peritoneal carcinomatosis with ascites, bilateral ovarian masses, and diffuse lymphadenopathy. Her presentation was determined to be concerning for breast cancer recurrence, and treatment with trastuzumab/pertuzumab was initiated.

Two months later, she developed worsening ascites. Cytology of paracentesis fluid revealed high-grade malignant cells (Fig. 2B) that were ER negative, PR positive (5–10%), and HER2 positive (3+). Additional immunohistochemistry revealed paired-box gene 8 (PAX-8), Wilms’ tumor 1 (WT-1) (Fig. 2C), and EMA expression. The cells were negative for GATA-binding protein 3 (GATA-3) and mammaglobin. This profile was consistent with cells from a high-grade Mullerian primary carcinoma. Considering these unexpected cytology findings, additional immunohistochemistry analysis was performed on the original left breast biopsy specimen, and similarly revealed PAX-8 and WT-1 positivity (Fig. 2C), with negative GATA-3 and mammaglobin expression. Combined WT-1 and PAX-8 expression is quite typical for Mullerian carcinoma but is essentially not seen in breast carcinomas.^4^ Together with her clinical presentation, these findings implicated that the cutaneous metastases to the breast were most likely from a primary Mullerian carcinoma rather than a breast carcinoma. Treatment with trastuzumab/pertuzumab was continued given HER2 expression. She died from the disease after 1 year.

## Discussion

Cutaneous metastasis to the breast is a rare phenomenon and may be challenging to diagnose, especially in patients who are at risk for developing multiple hormone receptor-altered malignancies due to *BRCA1/2* mutations.^3,4^ Hormone receptor status and HER2 expression may not successfully differentiate breast from other gynecologic tumors.^5^ WT-1 and PAX-8 expression, with concurrent negative mammaglobin and GATA-3 expression, are classically expressed in tumors of Mullerian origin, and can be beneficial to clarify the diagnosis.^4^ When evaluating an unusual breast rash, dermatologists might consider including a secondary metastatic malignancy on the differential, particularly if systemic symptoms are present. If there is concern for ovarian or gynecologic metastasis, analysis with PAX-8, WT-1, mammaglobin, and GATA-3 may be beneficial. Determining the origin of cutaneous metastases is critical and may lead to the diagnosis of an occult tumor, as in our case. Accurately determining the primary site that gives rise to metastases is required to ensure appropriate treatment and optimal therapeutic strategies are pursued.

## Conflicts of interest

None.

## Funding

None.

## Study approval

This study adhered to the recommendations and guidelines of the University of Michigan Medical School Institutional Review Board.

## Author contributions

ACH and RLC: Obtained interpreted the original immunohistochemistry photographs. GCH, ACH, RLC, and EAP: Prepared the figures and wrote the manuscript.

## Patient consent

The authors obtained written consent from the patient next of kin for use of the patient’s photographs and medical information to be published online and with the understanding that this information may be publicly available and discoverable via search engines. Patient consent forms are not provided to the journal but are retained by the authors.

## Acknowledgments

The authors would like to thank the patient and her family for use of photos and information.
